# Use of low-dose varenicline in patients who do not tolerate standard-dose varenicline: A longitudinal case series

**DOI:** 10.18332/tpc/194629

**Published:** 2025-03-14

**Authors:** Martha Swanson, Luisa C. Masclans, James M. Davis

**Affiliations:** 1 Duke Cancer Institute, Durham, United States; 2 Duke University School of Medicine, Durham, United States; 3 Department of Medicine, Duke University School of Medicine, Durham, United States

**Keywords:** smoking cessation interventions, recruitment feasibility, mobile applications, young adults, low socioeconomic status

## Abstract

**INTRODUCTION:**

Although varenicline tartrate is the most effective monotherapy for smoking cessation, the standard-dose (1 mg twice daily) is associated with adverse events: gastrointestinal, sleep-related, and mood-related. Lower doses have demonstrated similar efficacy with lower adverse event incidence. The purpose of this study was to determine whether patients who previously discontinued standard-dose varenicline due to adverse events could tolerate and benefit from low-dose varenicline.

**METHODS:**

We conducted a prospective longitudinal pilot study of 22 adult daily smokers in Durham NC, USA, in 2022. All participants previously discontinued standard-dose varenicline due to adverse events. These patients were prescribed either 0.5 mg twice daily for varenicline-related nausea or 1 mg in the morning for sleep problems. The primary outcome was change in self-reported adverse event severity (scale: 0–7). Secondary outcomes were smoking abstinence at 6-week follow-up and tolerance of the lower dose.

**RESULTS:**

Patients with intolerable nausea reported significant severity reduction (6.00 to 0.00; p<0.001) as did patients with intolerable vivid dreams (3.27 to 0.27; p=0.001). Smoking abstinence rates were 28.6% for 0.5 mg twice daily and 26.7% for 1 mg once daily. Low-dose varenicline tolerance was 81.8%.

**CONCLUSIONS:**

Patients who experience significant nausea with standard-dose varenicline may successfully make transition to a 0.5 mg low dose twice daily and those who experience vivid dreams to 1 mg varenicline in the morning. Treatment efficacy rates remained relatively high. This suggests a need for a future randomized controlled trial to establish low-dose varenicline as an approach for patients who do not tolerate the standard-dose varenicline.

## INTRODUCTION

### Pros and cons of varenicline tartrate

Smoking is the second leading cause of preventable mortality globally (after hypertension), causing 8 million annual deaths; 80% of the world’s 1.3 billion tobacco users reside in low- and middle-income countries^1^. In the United States, there are seven Food and Drug Administration (FDA) approved medications for use in smoking cessation (varenicline, bupropion, nicotine patch, gum, lozenge, inhaler, and nasal spray)^2^. Large population meta-analyses (n=157179) of randomized controlled trials show that varenicline, a nicotinic receptor partial agonist is significantly more effective than other monotherapies for smoking cessation in adults^3^. Unfortunately, varenicline use is frequently hindered by a high incidence of adverse events^4,5^. Most common adverse events fall into one of three categories: gastrointestinal (nausea – most common, vomiting, taste perversion, flatulence constipation); sleep problems (vivid dreams, nightmares, insomnia); and psychiatric (irritability, mood changes)^6-8^. While concerns regarding increased risk of suicidality and other severe psychiatric effects have been raised, large observational studies and a large randomized controlled trial (EAGLES Trial, n=8144) found that varenicline use was not associated with significantly higher incidence of moderate/severe psychiatric symptoms compared to placebo, bupropion or nicotine patch^6,9^.

### Efficacy of low-dose versus high-dose varenicline

Several comparative trials have shown that low-dose varenicline (0.5 mg twice daily or 1 mg once daily) has superior efficacy to placebo in adults, and one study showed essentially identical abstinence rates between standard and 0.5 mg twice daily varenicline treatment (46.5% and 46.4% abstinence, respectively, at 52 weeks)^7,10-12^. Perhaps the most compelling comparison comes from a 2016 meta-analysis by the Cochrane group comparing standard versus low-dose varenicline’s 52-week post-quit smoking abstinence rates in which standard-dose varenicline showed a very small advantage over low-dose varenicline (RR=1.25; 95% CI: 1.00–1.55, p=0.051)^8^. In summary, available data appear to show that standard-dose varenicline is likely marginally more effective than low-dose varenicline, with data from large meta-analyses remaining inconclusive.

### Adverse events from low-dose versus high-dose varenicline

Regarding adverse events, a 2016 meta-analysis found a significant association between use of standard-dose varenicline and nausea, insomnia, abnormal dreams and headache^8^. Individual studies have shown that lower doses of varenicline yielded numerically lower levels of nausea relative to standard-dose varenicline, based upon descriptive statistics^11,12^. Nides et al.^[Bibr CIT0011]11^ also found a lower incidence of sleep problems with a reduced varenicline dose. Oncken et al.^[Bibr CIT0007]7^ found that low-dose varenicline (0.5 mg twice daily, titrated up from 0.5 mg once daily) was not significantly associated with nausea relative to placebo, whereas full dose varenicline and non-titrated doses were significantly associated with nausea relative to placebo. In summary, available data show that low-dose varenicline likely induces a lower incidence of nausea and sleep problems relative to standard-dose varenicline, and titrated low-dose varenicline yields significantly lower incidence of nausea. Thus far, no published research has characterized how lower doses of varenicline affect patients that have previously experienced intolerable side effects at the standard-dose, a scenario which is likely in a clinical setting.

### Research question

Currently, available research shows that low-dose varenicline is likely similarly, or minimally less effective compared to standard-dose varenicline for smoking cessation and suggests that low-dose varenicline is associated with a lower incidence of adverse events^7,8,11,12^. Thus, it is reasonable to propose that low-dose varenicline might be an acceptable treatment choice for those who fail standard dosing. Thus far there is no study assessing this. Our study was designed to address this question in a naturalistic setting amongst a clinical population of patients with a history of intolerable adverse events taking full dose varenicline.

## METHODS

### Study design

We conducted a prospective longitudinal case series with 22 patients receiving treatment within the Duke Smoking Cessation Program, a group of medical and psychological providers in Durham, North Carolina that exclusively treat nicotine dependence in alignment with evidence-based practice in both outpatient and tele-health settings. Sample size was determined by the number of eligible patients who presented for care and gave consent to participate within a 6-month period. Inclusion criteria included: patients must be daily cigarette smokers, aged ≥18 years, with a history of intolerable adverse events (stopping varenicline due to adverse events) using standard-dose varenicline (1 mg twice daily), and willingness to use a lower dose. Patients were initially offered varenicline for a variety and sometimes multiple reasons including poor treatment outcomes, side effects from other medications (NRT, bupropion), or if they expressed interest in using varenicline. All patients had experienced some subjective meaningful treatment effect with varenicline in the past, but side effects had made varenicline intolerable. The study was conducted in patients only using varenicline with no other combination tobacco treatment pharmacotherapy. There was a wide range of time intervals between prior use of full dose varenicline and initiation of low-dose varenicline, with some patients having used standard-dose varenicline many years prior and others transitioning immediately after discontinuing standard-dose varenicline. All patients gave informed consent for study participation, and protected health information (PHI) was de-identified when entered. The study was designed to closely mimic standard clinical care, following program treatment procedures with no exclusion criteria. At baseline, all patients completed a 0–7 Likert scale questionnaire on the severity of six commonly reported adverse events during their previous use of standard-dose varenicline, from no symptoms at 0 to severe symptoms at 7. These were nausea, vomiting, vivid dreams, nightmares, irritability, and mood changes. Likert scale data on adverse event frequency were collected, but not reported here.

### Selection of dosing regimen for low-dose varenicline

Choice of dosing regimen for the study was based upon the nature of the adverse event experienced with varenicline. If participants reported a history of nausea with varenicline, they were prescribed varenicline 0.5 mg twice daily, decreasing the amount administered taken at once and overall. If patients reported a history of sleep problems with varenicline, they were prescribed varenicline 1 mg once each morning, with the goal of reducing the serum varenicline levels while sleeping. Given that the half-life of varenicline is 24 hours, serum levels of morning varenicline, should be modestly reduced by the end of the day^13^.

### Primary outcome

The primary outcome was change in mean adverse event severity after changing from standard-dose varenicline to low-dose varenicline based upon Likert scale questionnaires.

### Secondary outcomes

One secondary outcome was smoking cessation, defined as percentage of patients who had not smoked within the past week, assessed 6 weeks after starting low-dose varenicline. The other was medication tolerance, defined as the percentage of patients who continued taking low-dose varenicline 6 weeks after low-dose varenicline initiation.

### Statistical analysis

The primary outcome was change in the adverse events’ mean severity rating, rated on a 0–7 Likert scale between patients’ previous experience with the full-dose and the study-provided low-dose varenicline. Secondary outcomes included self-reported smoking abstinence at follow-up visits and tolerance of low-dose varenicline, defined as not discontinuing low-dose varenicline due to intolerable adverse events. Patients who did not get their low-dose varenicline prescription at their pharmacy, did not adhere to dosing instructions or did not attend enrollment visits, were not included in the analysis. Two tailed paired t-tests were used to compare changes in mean adverse event severity.

## RESULTS

### Sample

Over a 6-month period, 32 adult daily smokers seeking treatment at the Duke Smoking Cessation Program were found to have a history of varenicline discontinuation due to adverse events with willingness to use low-dose varenicline as a smoking cessation treatment. Of these, 10 were excluded. Three did not get the varenicline from their pharmacy. Six did not attend the study enrollment visit, and one did not take varenicline as prescribed. This yielded a sample of 22 participants with the following demographics: mean age of 53.9 years (SD=12.3); 59.1% female and 40.9% male; 63.6% White, 31.8% Black and 4.5% other race; mean lifetime number of cigarettes smoked per day was 20.3 (SD=10.2).

### Primary outcome: Adverse events related to low-dose varenicline

*History of nausea*

Seven patients with a history of intolerable nausea with standard-dose varenicline were prescribed varenicline 0.5 mg twice daily. These patients reported a significant reduction in nausea severity (6.00 to 0.00 on a 0–7 Likert scale; p<0.001) (Table 2).

*History of sleep problems*

Fifteen patients with a history of intolerable sleep-related adverse events (nightmares, vivid dreams) with standard-dose varenicline were prescribed 1 mg in the morning. These patients reported a significant reduction in vivid dreams (3.27 to 0.27 on a 0–7 Likert scale; p=0.001) and trend-level reduction in nightmares (2.13 to 0.0; p=0.053) (Table 1).

**Table 1:** Tolerability ^a^ of low-dose varenicline in smokers intolerant of standard-dose varenicline, longitudinal case series, outpatient clinic, Durham, USA, 2022 (N=22)

	*Dose: 0.5 mg twice daily* *(N=7)*			*Dose: 1 mg in the morning* *(N=15)*		
	*Prior Mean*	*Subsequent* *Mean*	*p*	*Prior Mean*	*Subsequent* *Mean*	*p*
Nausea	6	0	2.03E-09*	1.27	1.14	0.795
Vomiting	0.43	0	0.337	0.47	0	0.326
Vivid dreams	0	0	NA	**3.27**	**0.27**	0.001*
Nightmares	0	0	NA	2.13	0.33	0.053
Irritability	1	1	1	0.47	0	0.326
Mood changes	0	0	NA	0	0.47	0.326
Tolerated new dose (%)	71.4			86.7		
Smoking abstinence (%)	28.6			26.7		

a Mean intensity of adverse events before and after low-dose varenicline (1–7 Likert scale). BID: twice daily. qAM: every morning.

* p<0.05.

*History of psychiatric side effects*

Two patients reported history of 7/7 irritability with standard-dose varenicline. After starting low-dose varenicline, one remained at 7/7, the other reported 0/7 irritability. Further analysis was not conducted due to small sample size.

### Secondary outcomes

*Smoking abstinence on low-dose varenicline*

The self-reported smoking abstinence rate at clinical follow-up visits (mean 6 weeks post varenicline initiation) for patients prescribed 0.5 mg twice daily was 28.6% (95% CI: 8.2–64.1), for patients prescribed 1 mg daily was 26.7% (95% CI: 10.9–52.0), and for all patients was 27.3% (95% CI: 13.2–48.2) (Table 2).

**Table 2:** Tolerability and efficacy of low-dose varenicline by sex, race, age, and smoking history, longitudinal case series, outpatient clinic, Durham, USA, 2022 (N=22)

	*Total* *n (%)*	*Sex (Female)* *n (%)*	*African* *American* *n (%)*	*Age* *(years)* *Mean (SD)*	*Lifetime* *cigarettes/* day* *Mean (SD)*
Total	22 (100)	13 (59.1)	7 (31.8)	53.9 (12.2)	20.3 (10.2)
Stopped low-dose varenicline due to side effects	4 (18.2)	3 (75.0)	2 (50.0)	46.3 (15.6)	14.4 (6.6)
Tolerated full course of low-dose varenicline	18 (81.8)	10 (52.6)	5 (26.3)	55.6 (11.1)	21.6 (10.6)
Did not quit smoking on low-dose varenicline	16 (72.7)	9 (56.3)	7 (43.8)	52.4 (12.9)	20.0 (10.8)
Quit smoking on low-dose varenicline	6 (27.3)	4 (66.7)	0 (0)	57.7 (10.1)	21.3 (9.5)

* Lifetime cigarettes/day: self-reported average cigarettes smoked per day for the bulk of time spent smoking.

*Medication tolerance*

An analysis of all patients in the sample showed that 81.8% (95% CI: 61.5–92.7) tolerated low-dose varenicline, and 18.2% discontinued it due to adverse events (reporting chest pain, nightmares, irritability, and mood changes) (Table 2).

### Interpretation of main findings

Findings show that low-dose varenicline was associated with a significant reduction in nausea intensity for patients using 0.5 mg twice daily and a significant reduction in vivid dreams for patients using 1 mg each morning. The study sample of 2 patients who discontinued due to psychiatric adverse events was insufficient to assess effects of low-dose varenicline on psychiatric symptoms.

These study findings are clinically relevant because varenicline is the most effective smoking cessation monotherapy but confers a high rate of adverse events^3,5^, and low-dose varenicline has now been found to show efficacy that is similar to or slightly less effective than full dose varenicline^8^, with a lower incidence of adverse events^7^. This study addressees a very specific population – those who have already failed treatment with varenicline due to intolerable adverse events but who are willing to use it at a lower dose. The study found that low-dose varenicline was both tolerable and effective in these patients.

Quit rates were a secondary outcome in this study, and a larger sample size with a control group would be ideal to obtain more meaningful data on quit rates associated with low-dose varenicline. One question is if the observed quit rate of 27.3% is acceptable.

This is much higher than what would be expected in an unassisted quit attempt, with a real-life success rate of 4.5%, which doubles to 11% with any degree of professional support^14-16^. Furthermore, the quit rate observed in this study is similar to the absolute cessation rate of 27.6% observed with varenicline in a Cahill 2014 systematic review with meta-analysis of 3496 patients, albeit with a much smaller sample^17^. Given that these quit rates were seen amongst a population of patients who had already failed a trial of varenicline, such results are particularly encouraging.

### Limitations

Limitations of this study include a small sample size of 22 patients. While results are significant, the small sample sizes preclude generalizations about broad clinical applicability. A larger sample size would be necessary to generate more stable results. Additionally, this was an observational study without comparative groups that would be available in a randomized controlled study. Additionally, the primary comparison in this study is between two symptom scores, one based on historical memory of experience with standard-dose varenicline, and the other from a more recent low-dose varenicline trial. The historical reports of past adverse events with varenicline are subject to disproportionate recall bias, in which bias in reporting of past standard-dose adverse events would differ from reporting biases for more recent low-dose-related adverse events^18^.

## CONCLUSIONS

This case series provides preliminary evidence to suggest that low-dose varenicline may be a tolerable and effective treatment for patients who experience gastrointestinal or sleep-related adverse events with standard-dose varenicline. Results suggest the need for a future comparative trial to establish findings with greater certainty.

## Data Availability

The data supporting this research are available from the authors on reasonable request.
